# Supplementary material for: Inhibitory Effects of Epipolythiodioxopiperazine Fungal Metabolites on Isocitrate Lyase in the Glyoxylate Cycle of Candida albicans
Source: Mar Drugs. 2021 May 22;19(6):295. doi: 10.3390/md19060295 (PMC8224697; doi:10.3390/md19060295)
Supplement: Supplementary file 1 [file marinedrugs-19-00295-s001.zip › marinedrugs-1218500-supplementary.pdf]

Supporting Information

Inhibitory Effects of Epipolythiodioxopiperazine Fungal Metabolites on Isocitrate Lyase in the Glyoxylate Cycle of *Candida albicans*

Ji-Yeon Hwang [1], Beomkoo Chung [2], Oh-Seok Kwon [1], Sung Chul Park [1], Eunji Cho [2], Dong-Chan Oh [1], Jongheon Shin [1,*] and Ki-Bong Oh [2,*]

[1] Natural Products Research Institute, College of Pharmacy, Seoul National University, Seoul 08826, Korea

[2] Department of Agricultural Biotechnology, College of Agriculture and Life Sciences, Seoul National University, Seoul 08826, Korea

Contents

Figure S1 The ^{1}H NMR spectrum of secoemestrin C (**1**) (400MHz, $CDCl_3$) S3
Figure S2 The ^{13}C NMR spectrum of secoemestrin C (**1**) (100MHz, $CDCl_3$)------S4
Figure S3 The ^{1}H NMR spectrum of dethiosecoemestrin (**2**) (400MHz, $CDCl_3$) S5
Figure S4 The ^{13}C NMR spectrum of dethiosecoemestrin (**2**) (100MHz, $CDCl_3$) S6
Figure S5 The ^{1}H NMR spectrum of emestrin (**3**) (800MHz, DMSO-d_6)------S7
Figure S6 The ^{13}C NMR spectrum of emestrin (**3**) (200MHz, DMSO-d_6)------S8
Figure S7 The ^{1}H NMR spectrum of emestrin B (**4**) (600MHz, $CDCl_3$)------S9
Figure S8 The ^{13}C NMR spectrum of emestrin B (**4**) (150MHz, $CDCl_3$)------S10
Table S1 The Deviations with literature of ^{13}C NMR Chemical shifts of **1-4** S 1 1

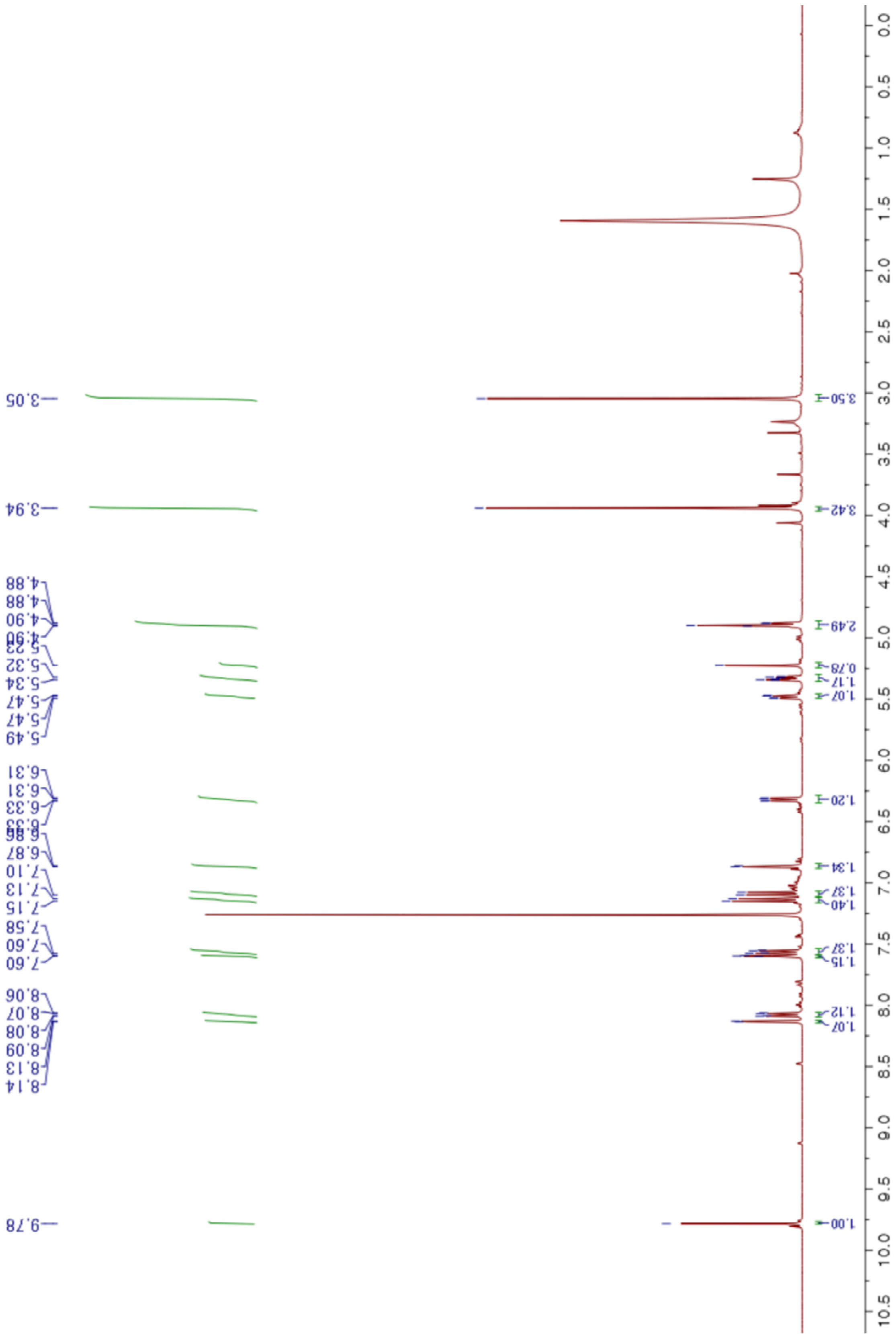

Figure S1. The ^{1}H NMR spectrum of secoemestrin C (**1**) (400MHz, $CDCl_3$)

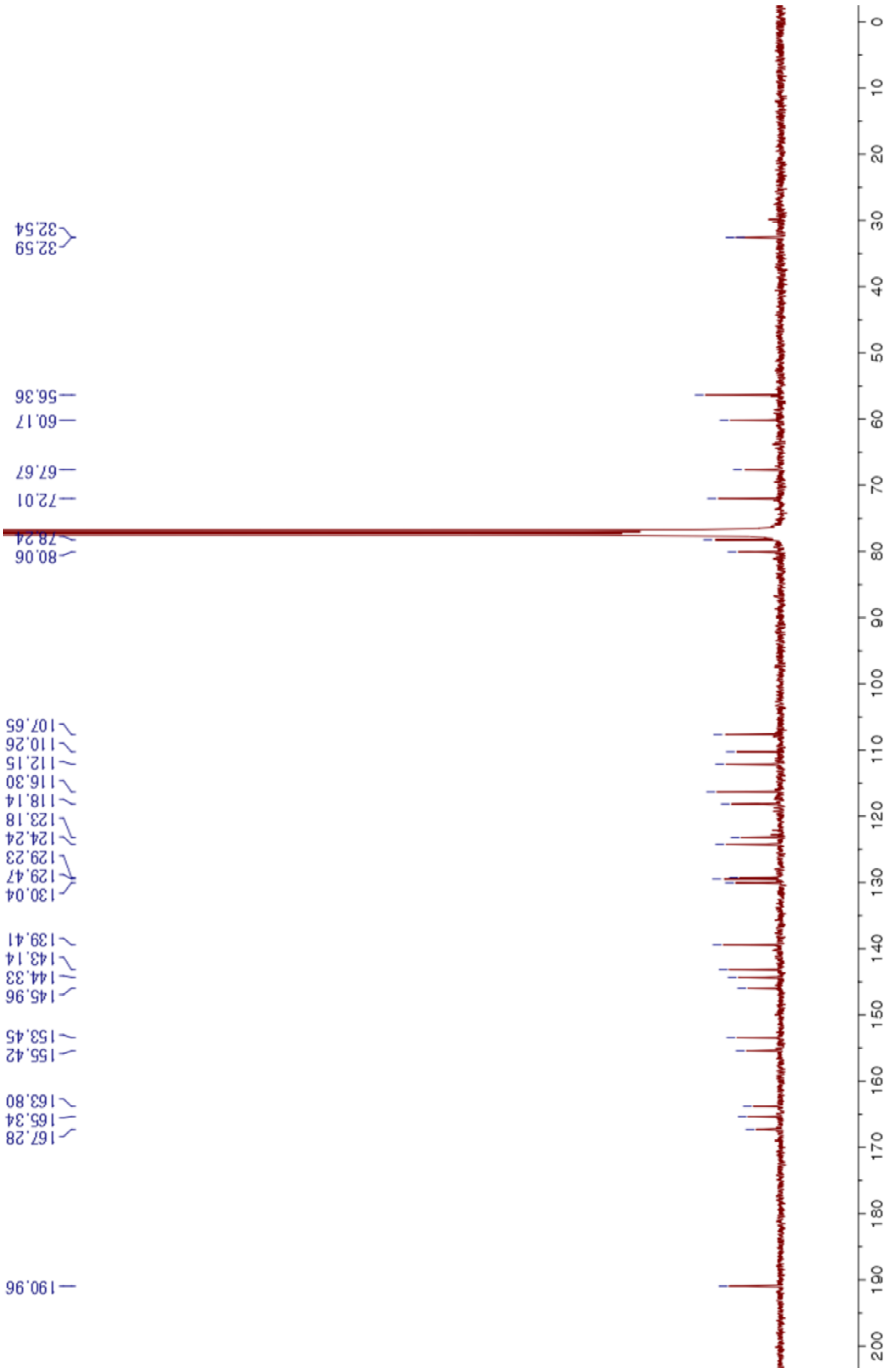

Figure S2. The ^{13}C NMR spectrum of secoemestrin C (**1**) (100MHz, $CDCl_3$)

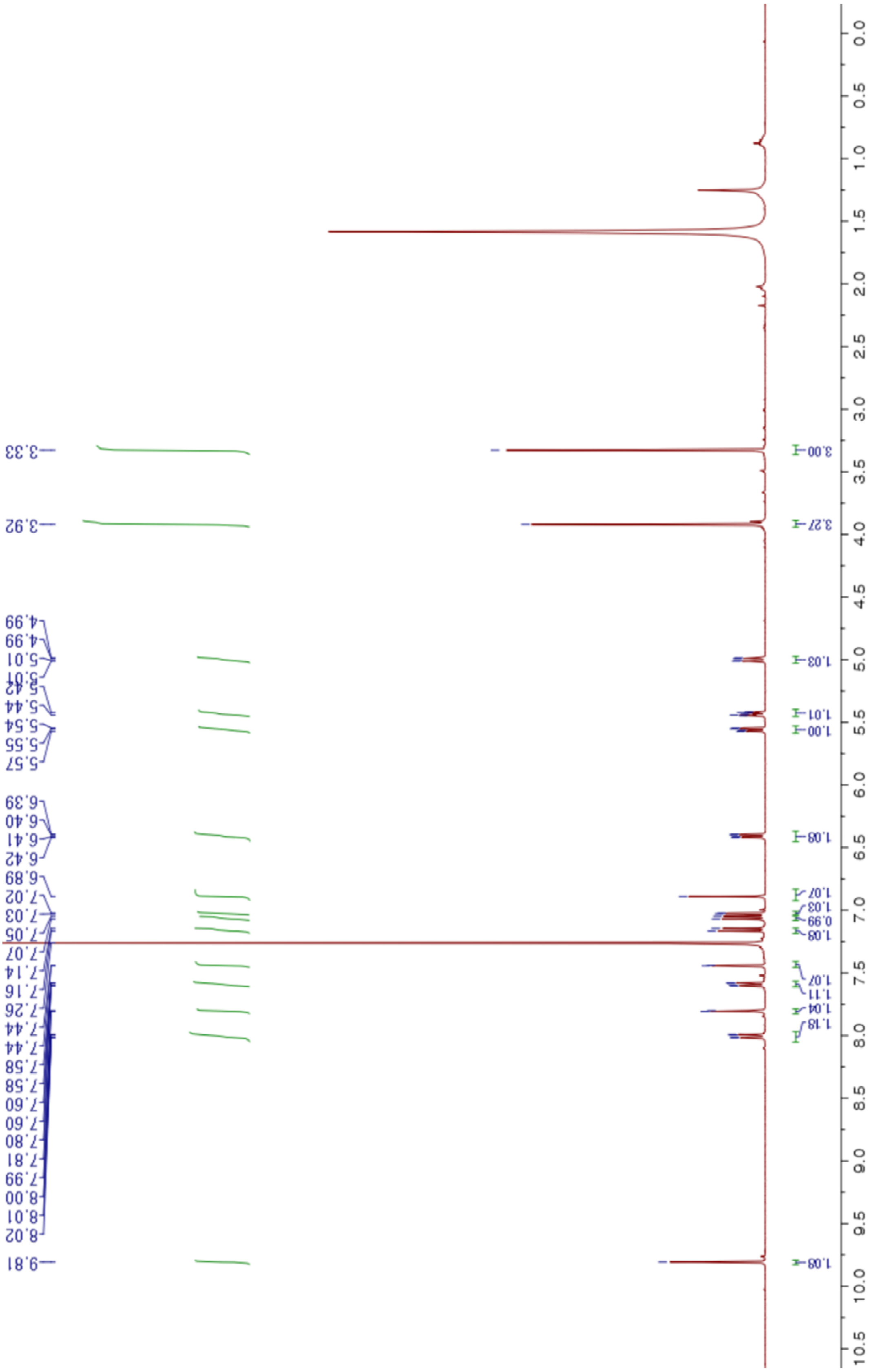

Figure S3. The ^1H NMR spectrum of dethiosecoemestrin (**2**) (400MHz, $CDCl_3$)

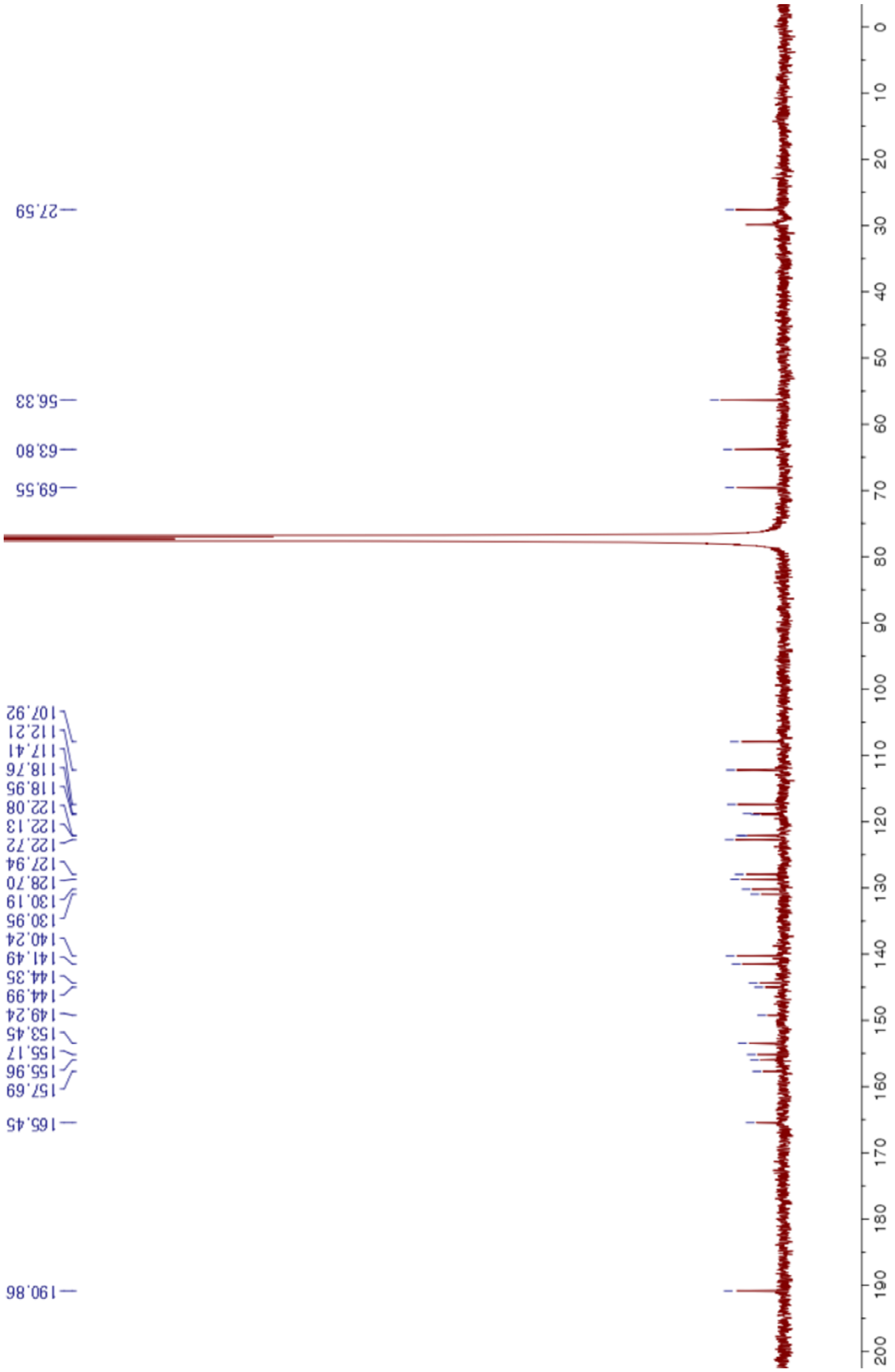

Figure S4. The ^{13}C NMR spectrum of dethiosecoemestrin (**2**) (100MHz, $CDCl_3$)

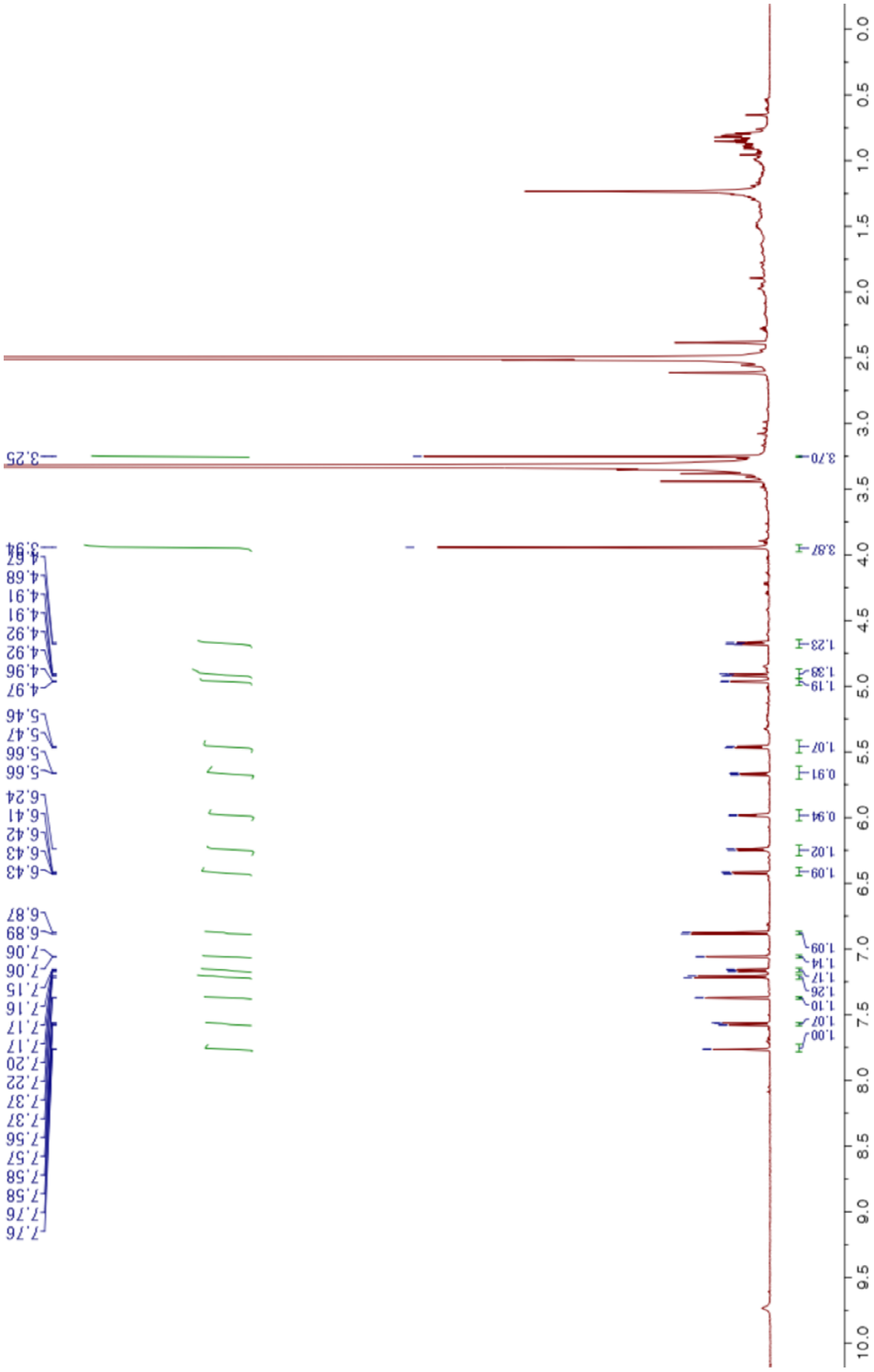

Figure S5. The ^{1}H NMR spectrum of emestrin (**3**) (800MHz, DMSO-d_6)

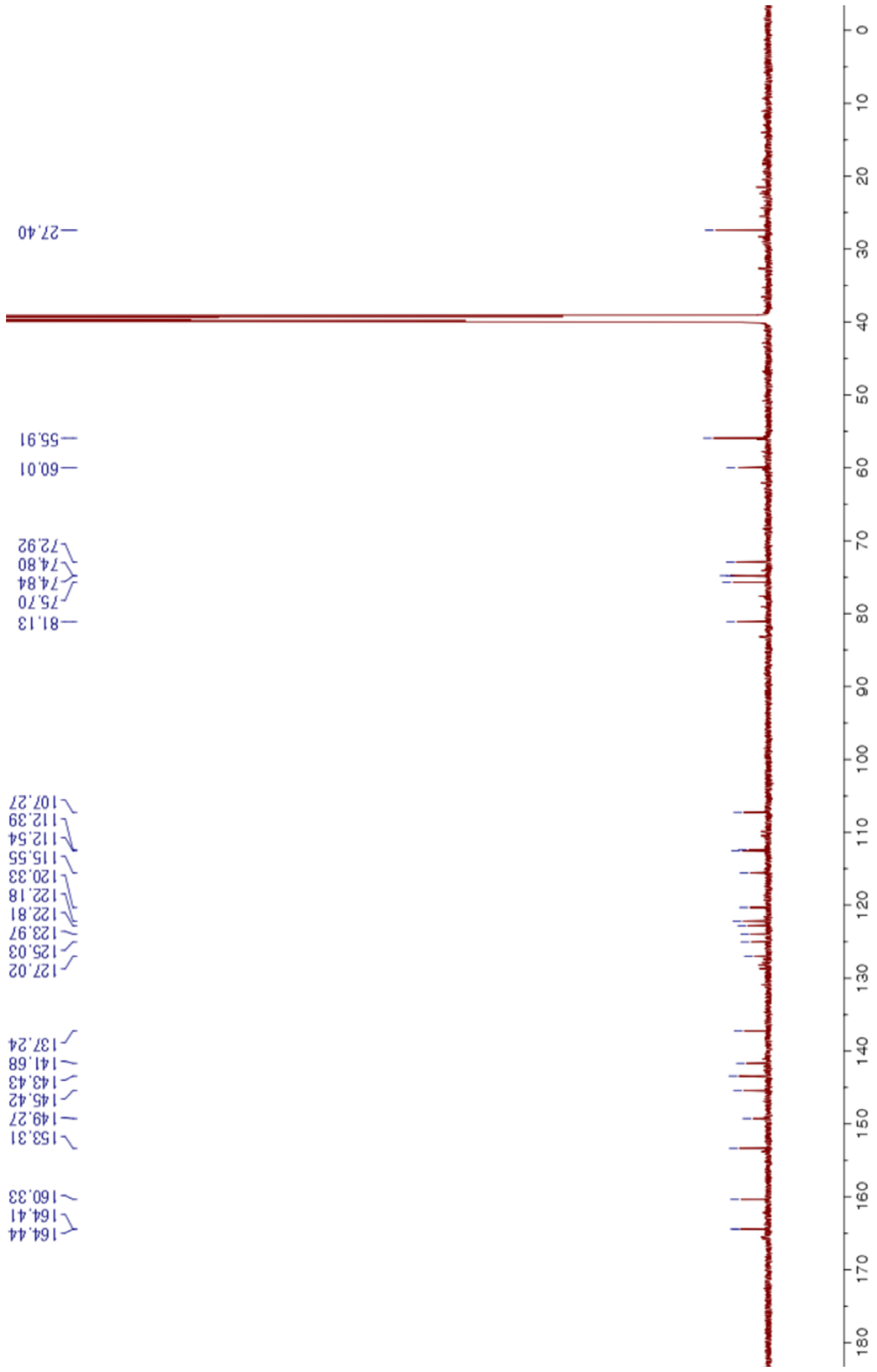

Figure S6. The ^{13}C NMR spectrum of emestrin (**3**) (200MHz, DMSO-d_6)

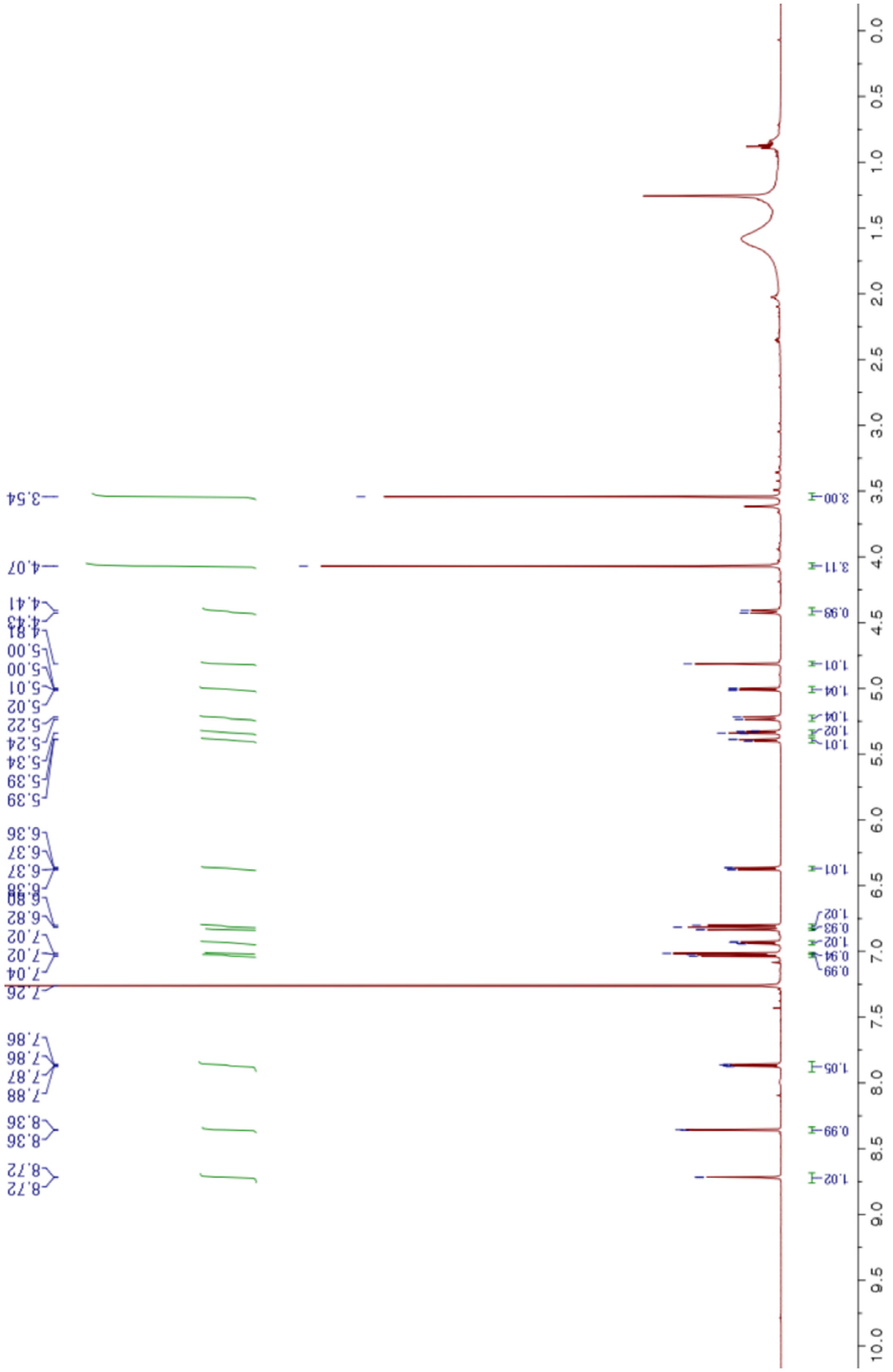

Figure S7. The ^{1}H NMR spectrum of emestrin B (**4**) (600MHz, $CDCl_3$)

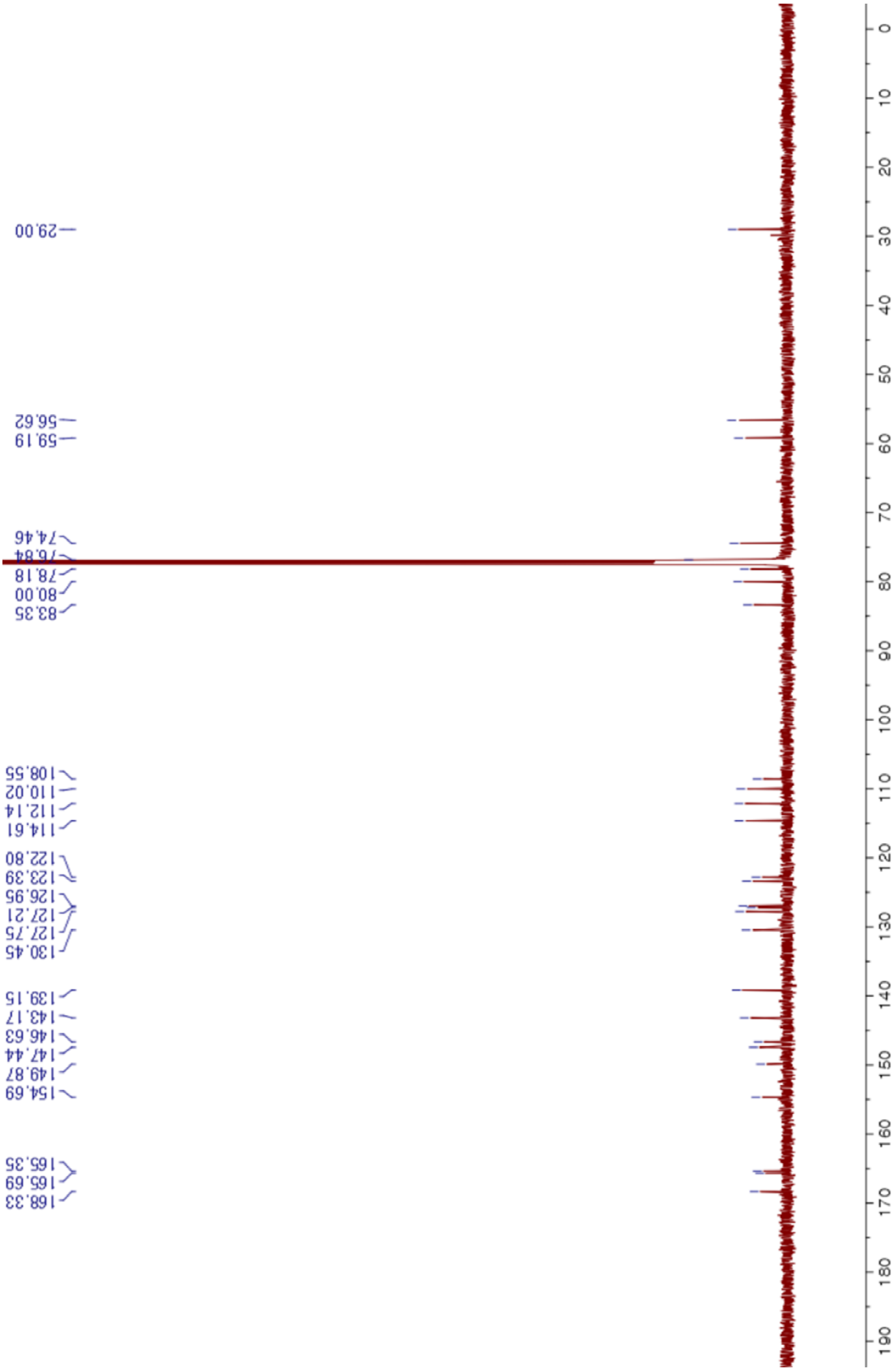

Figure S8. The ^{13}C NMR spectrum of emestrin B (**4**) (150MHz, $CDCl_3$)

Table S1. The Deviations with literature of ^{13}C NMR Chemical shifts **1-4**

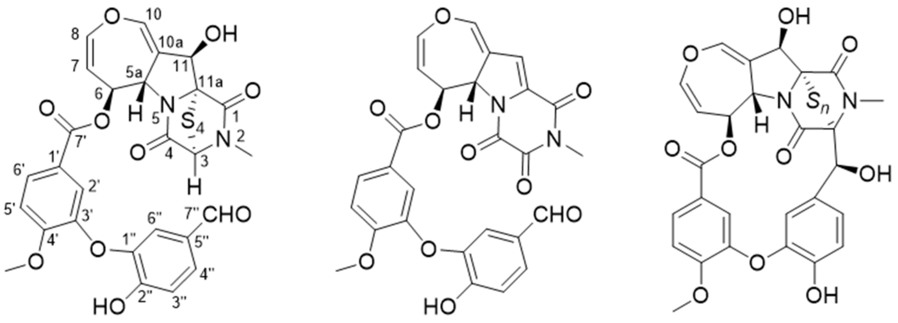

1

2

3 *n=2*

4 *n=3*

	secoemestrin C (**1**)			dethiosecoemestrin			emestrin (**3**)			emestrin B (**4**)		
No.	Ref.[1]	**1**	Dev	Ref.[2]	**2**	Dev	Ref.[3]	**3**	Dev	Ref.[4]	**4**	Dev
1	166.7	167.3	-0.6	155.9	155.9	0	164.3	164.4	-0.1	165.7	165.7	0
2-NMe	32.3	32.6	-0.3	27.3	27.6	-0.3	27.3	27.4	-0.1	29.0	29.0	0
3	68.6	67.7	0.9	157.5	157.7	-0.2	75.6	75.7	-0.1	83.3	83.4	-0.1
4	164.3	163.8	0.5	149.2	149.2	0	160.6	160.3	0.3	165.4	165.4	0
5a	60.2	60.2	0	63.8	63.8	0	59.9	60.0	-0.1	59.2	59.2	0
6	72.7	72	0.7	69.4	69.6	-0.2	72.9	72.9	0	74.9	74.5	0.4
7	108.5	107.7	0.8	107.8	107.9	-0.1	107.2	107.3	-0.1	110.0	110.0	0
8	140.0	139.4	0.6	140.2	140.2	0	137.2	137.2	0	139.2	139.2	0
10	142.3	143.1	-0.8	141.2	141.5	-0.3	141.7	141.7	0	143.2	143.2	0
10a	113.2	110.3	2.9	118.9	118.9	0	112.4	112.5	-0.1	108.5	108.6	-0.1
11	78.6	78.2	0.4	122.2	122.1	0.1	74.8	74.8	0	76.8	76.8	0
11a	81.1	80.1	1.0	131.2	130.9	0.3	81.0	81.1	-0.1	78.1	78.2	-0.1
1′	123.8	124.2	-0.4	122.2	122.7	-0.5	122.2	122.2	0	122.8	122.8	0
2′	122.9	123.2	-0.3	122.2	122.1	0.1	123.9	123.9	0	130.5	130.5	0
3′	145.4	144.3	1.1	144.3	144.4	-0.1	143.4	143.4	0	146.6	146.6	0
4′	156.2	155.4	0.8	155.2	155.2	0	148.9	149.3	-0.4	149.9	149.9	0
4′-OMe	56.7	56.4	0.3	56.2	56.3	-0.1	55.8	55.9	-0.1	56.7	56.6	0.1
5′	113.2	112.2	1.0	112.2	112.2	0	115.4	115.5	-0.1	112.1	112.1	0
6′	129.0	129.5	-0.5	128.6	128.7	-0.1	124.9	125.0	-0.1	127.8	127.8	0
7′	165.8	165.3	0.5	165.2	165.5	-0.3	164.4	164.4	0	168.3	168.3	0
1″	146.5	145.9	0.6	145.1	144.9	0.2	145.3	145.4	-0.1	147.4	147.4	0
2″	154.6	153.5	1.1	153.3	153.5	-0.2	153.3	153.3	0	154.7	154.7	0
3″	117.7	116.3	1.4	117.2	117.4	-0.2	112.3	112.4	-0.1	114.6	114.6	0
4″	128.5	129.2	-0.7	127.6	127.9	-0.3	122.7	122.8	-0.1	126.9	126.9	0
5″	130.8	130.1	0.7	130.1	130.2	-0.1	127.1	127.0	0.1	127.2	127.2	0
6″	118.5	118.1	0.4	118.7	118.8	-0.1	120.4	120.3	0.1	123.4	123.4	0
7″	190.9	190.9	0	190.5	190.9	-0.4	74.8	74.8	0	79.9	80.0	-0.1

[1] Ooike, M.; Nozawa, K.; Kawai, K.-I. An epitetrathiodioxopiperazine related to emestrin from *Emericella foveolata*. *Phytochemistry* **1997**, *46*, 123–126.

[2] Seya, H.; Nozawa, K.; Udagawa, S.-I.; Nakajima, S.; Kawai, K.-I. Studies on fungal products. IX. dethiosecoemestrin, a new metabolite related to emestrin, from *Emericella striata*. *Chem. Pharm.*

Bull. **1986**, *34*, 2411–2416.
[3] Seya, H.; Nozawa, K.; Nakajima, S.; Kawai, K.-I.; Udagawa, S.-I. Studies on fungal products. Part 8. Isolation and structure of emestrin, a novel antifungal macrocyclic epidithiodioxopiperazine from *Emericeella striata*. X-Ray molecular structure of emestrin. *J. Chem. Soc. Perk. T. 1* **1986**, *1986*, 109–116.
[4] Nozawa, K.; Udagawa, S.; Nakajima, S.; Kawai, K. Studies on fungal products. XIV. emestrin B, a new epitrithiodioxopiperazine, from *Emericella striata*. *Chem. Pharm. Bull.* **1987**, *35*, 3460–3463.
